# Acute and timing effects of beta-hydroxy-beta-methylbutyrate (HMB) on indirect markers of skeletal muscle damage

**DOI:** 10.1186/1743-7075-6-6

**Published:** 2009-02-04

**Authors:** Jacob M Wilson, Jeong-su Kim, Sang-rok Lee, John A Rathmacher, Brett Dalmau, J Derek Kingsley, Heather Koch, Anssi H Manninen, Raz Saadat, Lynn B Panton

**Affiliations:** 1 Department of Nutrition, Food & Exercise Sciences, Florida State University, Tallahassee, FL, USA; 2 Department of Animal Sciences, Iowa State University, Ames, IA, USA; 3 Manninen Nutraceuticals Oy, Oulu, Finland

## Abstract

**Background:**

While chronic β-Hydroxy β-Methylbutyrate (HMB) supplementation (≥ 2 wk) lowers exercise induced muscle damage, its acute or timing effects have not been examined. The purpose of this study was to investigate the acute and timing effects of oral HMB supplementation on serum creatine kinase (CK), lactate dehydrogenase (LDH), muscle soreness, and maximal voluntary contraction (MVC).

**Methods:**

Sixteen non-resistance trained men (22 ± 2 yrs) were assigned to HMB-Pre or HMB-Post groups. In a crossover design, all subjects performed 55 maximal eccentric knee extension/flexion contractions on 2 occasions on either the right or left leg. HMB-Pre (N = 8) randomly received 3 grams of either a placebo or HMB before and a placebo after exercise. HMB-Post (N = 8) received a placebo before and either 3 grams of HMB or a placebo after exercise. Muscle damage tests were recorded before, at 8, 24, 48, and 72 hrs post exercise.

**Results:**

There was a reduction in MVC and an increase in soreness in the quadriceps and hamstrings following exercise (*p *< 0.001). Although HMB-Pre approached significance in attenuating soreness for the quadriceps (*p *= 0.07), there was no time × group effect. Serum indices of damage increased, peaking at 48 hrs for CK (773%) (*p *< 0.001) and 72 hrs for LDH (180%) (*p *< 0.001). While there were no time × group effects of HMB on CK and LDH, post hoc analysis revealed that only HMB-Pre showed no significant increase in LDH levels following exercise.

**Conclusion:**

Our findings suggest no clear acute or timing effects of HMB supplementation. However, consuming HMB before exercise appeared to prevent increases in LDH.

## Introduction

Oral administration of the leucine metabolite, beta-hydroxy beta-methylbutyrate (HMB), has been associated with increased lean body mass (LBM) [1,2], isometric [1], isokinetic [1], and dynamic strength [3]. Moreover, these positive effects of HMB supplementation have generalized across young [4], elderly [5], untrained [1,3], trained [6,7], and clinically cachexic conditions [8].

HMB is presently thought to at least partly exert its effects via increasing the availability of substrate for *de novo *cholesterol synthesis within skeletal muscle [3,9]. Specifically, HMB is converted to 3-hydroxy-3-methylglutaryl-coenzyme A (HMG-CoA) with a subsequent increase in the activity of the rate limiting enzyme for cholesterol synthesis HMG-CoA reductase [3,9]. The result is an overall reinforcement of the sarcolemma as well as the provision of valuable substrate for its repair following muscle damaging and or injurious exercise [3,9]. This is evidenced by studies demonstrating that HMB leads to decreased markers of muscle damage following mechanically strenuous exercise included lower levels of creatine kinase (CK) [3,4], lactate dehydrogenase (LDH) [10], 3-methylhistidine (3-MH) [3], urine urea nitrogen and plasma urea [2], and muscular soreness [4].

The effectiveness and generalizability of HMB supplementation merits further research into how to optimize its administration. Currently, the main variable examined has been the optimal dosage of HMB supplementation [1,3], which appears to be 3 grams per day [1,3]. However, there are no studies that have examined the acute effects of HMB administration (e.g its administration the day of exercise), as studies thus far chronically load the supplement (≥ 2 weeks) [3,6,11-13]. Moreover, if HMB does exert acute effects, we are unaware of research investigating if there is an advantage to consuming the supplement prior to as compared to after exercise (timing effects). Based on findings demonstrating that the timing of ingestion appears to be an important element in recovery from exercise for a number of nutrients and nutrient derivatives [14-16], we feel this is a worthy question of interest. Therefore, the purpose of this experiment was to investigate the acute and timing effects of an HMB supplement on indices of muscle damage and performance following a unilateral eccentrically based muscle damaging protocol in untrained subjects. For the reason that consuming HMB prior to exercise is thought to allow its incorporation into muscle tissue before the strenuous event, and because blood flow directed toward muscle tissue during exercise would enhance this effect, we hypothesized that consuming HMB prior to exercise would attenuate indices of muscle damage relative to both a placebo, and consumption of HMB after exercise.

## Methods

### Subjects

Sixteen healthy college-aged men (22 ± 2 yrs) were recruited for the study. Subjects could not have participated in a resistance exercise program or taken nutritional supplements for at least six months prior to data collection. Subjects also could not participate if they smoked, had high resting blood pressure (≥ 140/90 mmHg) or were currently taking anti-inflammatory agents. Screening for the above criteria was obtained by phone or in person prior to any testing. Each subject signed an informed consent approved by the University Institutional Review Board before participating in the study.

### Experimental design

On the first visit subjects reported to the laboratory after a 12-hour fast. Body composition was measured using the sum of 3 skinfolds (chest, abdomen, and thigh) following the procedures of Jackson and Pollock [17]. Baseline muscle soreness of the quadriceps and hamstrings was evaluated using the visual analogue scale (VAS). Reliability scores for the VAS have been reported as high at *r *= 0.97 for assessing soreness [18]. A baseline blood draw was taken to measure serum CK and LDH levels. The first treatment, either placebo (rice maltodextrin) or HMB supplement was administered in 12 individual gelatin capsules (3 grams) after the first 2 tests were completed. Following ingestion of the capsules, the subjects sat in the laboratory for 60 minutes. This duration was selected based on the rationale that a 3 gram bolus of HMB appears to peak in plasma 60 minutes after ingestion [19]. A second reason was based on the premise that HMB may interact with muscle in a similar manner to leucine [20]. After administration of leucine containing essential amino acid mixture, the rate of incorporation of labeled leucine into mixed muscle protein rapidly rises after 30 minutes, reaching peak values at 60 minutes before returning to baseline at 120 minutes [21]. Therefore, it was reasoned that a 60-minute interval would allow ample incorporation of HMB into skeletal muscle tissue. After the 60-minute period the subjects underwent an isometric strength test using a maximal voluntary contraction (MVC) of the quadriceps and hamstrings [16], in which reliability (test/retest) in our laboratory is reported high when using a 1 week interval (*r *= 0.99, N = 8). Following this test, the subjects performed an eccentric exercise bout. Immediately after the completion of the exercise protocol, the subjects ingested another 12 pills of either placebo or HMB. All measurements used before the eccentric exercise bouts were repeated at eight, 24, 48, and 72 hours after the eccentric exercise bout.

### Control of diet and exercise

All subjects were required to keep a record of their diet (all food and beverages) for 48 hours prior to and during the first experiment participated in. The dietary log was then given to the subject with instructions to replicate the food consumption for 48 hours prior to and during the second randomly assigned experiment [22]. In addition, subjects were told to refrain from taking any anti-inflammatory dietary supplements or medications to prevent any further nutritional or drug-related protection against the exercise-induced muscle damage. These restrictions were enforced 48 hours before and during the testing period. Finally, subjects were to keep activity to a minimum, and to not perform any strenuous exercise 72 hours before and during the testing period.

### Supplementation and resistance exercise protocol

Subjects were randomly assigned to either pre (n = 8) or post (n = 8) exercise HMB conditions. The MVC and muscle damaging exercise protocol was adapted from White et al. [16] in which all subjects performed 3 maximal isometric contractions of the hamstrings and quadriceps followed by 55 maximal eccentric unilateral knee extension/flexion contractions on 2 separate occasions using the Biodex™ (Shirley, New York) isokinetic machine, performed on the dominant or non-dominant leg in a counter-balanced crossover design. HMB-Pre (n = 8) received 3 grams of HMB before and a placebo after exercise, or a placebo before and after exercise in the counter-balanced crossover design separated by 2 weeks. HMB-Post (n = 8) received a placebo before and 3 grams of HMB after exercise, or a placebo before and after exercise in the counter-balanced crossover design again 2 weeks later. For the MVC protocol, the subject's upper and lower body was restricted with shoulder and leg straps to help isolate the exercising leg. Subjects completed ten sub-maximal concentric/concentric contractions of the quadriceps and hamstrings of the dominant or nondominant leg for a warm up. They were then tested for the MVC 3 minutes after the completion of the warm up. All testing for MVC was performed with the subject's knee joint placed at a 60° angle. Subjects alternated 5-second maximal isometric contractions separated by 30-second rest of the knee extensors and flexors for a total of 3 repetitions per contraction. The highest values were then recorded. After 3 minutes of rest, the subjects performed the eccentric protocol which was divided into 1 set of 5 repetitions followed by 5 sets of 10 repetitions. During the eccentric protocol the Biodex™ was set through a range of motion of 1.75 rad [100°], performed at an angular velocity of 1.05 rad/s [60°/s].

### Creatine kinase and lactate dehydrogenase

Five mL of blood was taken from an antecubital vein using sterile venipuncture techniques. Blood was collected in EDTA coated tubes and centrifuged for 10 minutes at 3200 rpm. Samples were separated and stored at -80°C until analysis. Serum CK and LDH activity were measured in duplicate using an enzymatic assay kit (StanBio Laboratories; Borne, Texas) after all samples had been collected. Our coefficient of variation between duplicates was less than 1%.

### Statistical analysis

Sixteen subjects were selected as this number allowed us to have 8 individuals placed in the HMB-Pre condition, and 8 to be placed in the HMB-Post condition. Our rationale for subject size was based on a study by Van Someren et al. [23]. In a randomized cross over design similar to our study, these investigators found that 2 weeks of HMB supplementation in 8 untrained subjects was able to significantly decrease serum CK, soreness, and the decline seen in MVC following an eccentric muscle damaging protocol. Using the equation: effect size (ES) = (experimental mean-control mean)/the pooled standard deviation of peak serum CK levels (μl), the study by Van Someren et al. had an ES of 1.4 = [(315-154)/116 μl]. Based on an alpha level of 0.05, a power of 80 and an effect size of 1.4, a total of 14 subjects were needed for the study.

A 3 × 5 [condition (HMB-Pre, HMB-Post, and placebo) × time (0, 8, 24, 48, and 72 hrs)] repeated Analyses of Variance (ANOVA) was used to test for significance between MVC values and VAS scores. A 3 × 4 [condition × time (8, 24, 48, and 72 hrs)] repeated ANOVA was used to test for significance for CK and LDH values which were normalized to a percentage. A Tukey HSD post hoc test was used to locate significance between time points if there was a main effect of time. All significance was accepted at *p *≤ 0.05. All statistical procedures were carried out on Statistica (StatSoft^®^, Tulsa, OK, USA).

## Results

### Subject characteristics

There were no differences between groups for age, body weight, height, percent body fat or lean mass (Table 1). All subjects verbally confirmed that they had not been involved in resistance exercise for at least six months and were not taking any dietary supplements or anti-inflammatory agents prior to the study. Of the 16 subjects who met screening criteria, none dropped out of the study, and all completed the entire protocol as described.

**Table 1:** Subject characteristics (N = 16)

**Variables**	**HMB-Pre (n = 8)**	**HMB-Post (n = 8)**
Age (yrs)	22 ± 2.0	22 ± 2.0

Height (cm)	177.0 ± 5.0	178 ± 4.0

Weight (kg)	72.0 ± 10.6	72.0 ± 9.0

Body Fat %	12.6 ± 6.0	13.2 ± 4.0

Values are means ± standard deviation.

* *p *< 0.05, significantly different from 0 time point

HMB pre exercise (HMB-pre), received supplement before exercise and placebo after; HMB post exercise (HMB-post), received supplement after exercise and placebo before exercise.

### Maximal Voluntary Contraction

**Figure 1:**
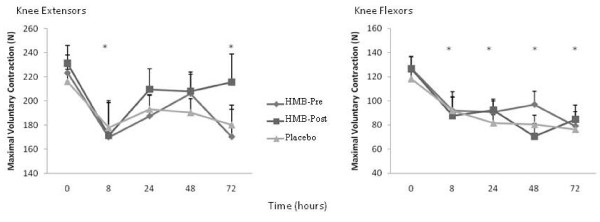
**Peak torque of maximal voluntary contraction (MVC) for the knee extensors and flexors across the 72 hours**. Values are means ± standard errors, * *p *≤ 0.05, significantly different from 0 time point, there were no differences among conditions. HMB-Pre, received HMB before exercise and placebo after; HMB-Post, received HMB after exercise and placebo before exercise; Control, received a placebo before and after exercise; N, newtons.

Baseline MVC scores for both the quadriceps and hamstrings were similar for all 3 conditions (Figure 1). There was no condition by time interaction for MVC values for either the quadriceps or hamstrings, however there were significant time effects for MVC in both the quadriceps (F_(4,116) _= 5.0, *p *≤ 0.05, ES = 0.15) and hamstrings (F_(4,116) _= 13.6, *p *≤ 0.05, ES = 0.30). After the exercise bout, MVC scores were significantly lower for all time points compared to the pre-exercise values (Figure 1), with the greatest declines occurring at 8 hrs in the quadriceps (-22%) and at 48 hrs in hamstrings (-38%) with no acute or timing differences observed with HMB supplementation.

### Muscle soreness

**Figure 2:**
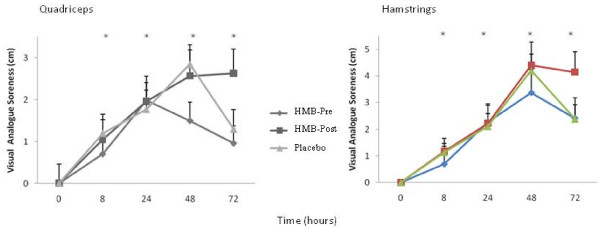
**Visual analogue soreness across the 72 hours for the quadriceps and hamstrings**. Values are means ± standard errors. * *p *≤ 0.05, significantly different from 0 time point, there were no differences among conditions. HMB-Pre, received HMB before exercise and placebo after; HMB-Post, received HMB after exercise and placebo before exercise; Control, received placebo before and after exercise.

Pre-exercise values were not different among conditions for muscle soreness (Figure 2). While the HMB-Pre condition approached significance in lowering quadriceps soreness (*p *= 0.07) and visually appeared to decrease soreness for hamstrings (Figure 2), there were no condition by time interactions found. However, there were main time effects for both the quadriceps (F_(4,116) _= 17, *p *≤ 0.05, ES = 0.37) and hamstrings (F_(4,116) _= 17, *p *≤ 0.05, ES = 0.53) soreness. After the exercise bout, soreness values were significantly elevated for all time points compared to the pre-exercise values (Figure 2, Table 1.0). Quadriceps soreness peaked at 24 h for HMB-Pre (2.0 cm), and at 48 h for both HMB-Post (2.6 cm) and placebo (2.8 cm) conditions (Figure 2), while hamstring soreness peaked at 48 h for HMB-Pre (3.4 cm), HMB-Post (4.4 cm), and placebo (4.2 cm) conditions (Figure 2).

### Changes in serum CK and LDH

**Figure 3:**
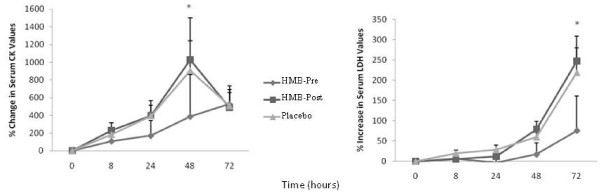
**Percent change in serum creatine kinase (CK) and lactate dehydrogenase (LDH) values across the 72 hours**. Values are means ± standard errors. * *p *≤ 0.05, significantly different from 0 time point, there were no differences among conditions. HMB-Pre, received HMB before exercise and placebo after; HMB-Post, received HMB after exercise and placebo before exercise; Control, received placebo before and after exercise.

There were no differences in serum CK values among the 3 conditions at baseline. While visually HMB-Pre appeared to decrease the rise in serum CK following exercise (Figure 3), there was no condition by time interaction after the exercise bout. However, there was a significant time effect (F_(3,87) _= 6.6, *p *≤ 0.05, ES = 0.20). A significant percent increase in CK was found for all 3 groups peaking at 48 hrs for both placebo (906%)(*p *< 0.001) and HMB-Post (1,000%)(*p *< 0.001) conditions, and at 72 hrs (508 %) for the HMB-Pre condition. The supplement or timing of ingestion had no significant effect on serum CK at any time points. There were no differences in LDH values among the 3 conditions at baseline. While there was a significant time effect for % increase in serum LDH (F_(3,87) _= 14.1, *p *≤ 0.05, ES = 0.33) (Figure 3), there was no time × group interaction. Post hoc analysis revealed that LDH values significantly increased in both the placebo (219%, 72 hrs) and HMB-Post (247%, 72 hr) groups (Table 2). However, there were no significant increases in LDH values in the HMB-Pre condition (Table 2, Figure 3).

**Table 2:** Values for CK, MVC, and VAS for the three supplement groups

**Variables**		**0**	**8 hrs**	**24 hrs**	**48 hrs**	**72 hrs**
MVC Knee Extensors	Control	216 ± 10	178 ± 29*	193 ± 17	191 ± 16	180 ± 22*

(N)	Pre	224 ± 15	170 ± 28*	188 ± 17	206 ± 16	165 ± 22*

	Post	231 ± 15	171 ± 28*	210 ± 16	208 ± 15	215 ± 23


MVC Knee Flexors	Control	118 ± 7	92 ± 11	82 ± 6	80 ± 8	76 ± 8

(N)	Pre	127 ± 10	92 ± 16	91 ± 9	97 ± 11	79 ± 12*

	Post	127 ± 12	88 ± 13	92 ± 11*	71 ± 12*	85 ± 14*


VAS Quadriceps	Control	0.0 ± 0.0	2.0 ± 0.4	2 ± 0.4	2.6 ± 1.4*	1.0 ± 0.4

(cm)	Pre	0.0 ± 0.0	0.7 ± 0.5	1.9 ± 1.0*	1.5 ± 1.0	0.9 ± 1.0

	Post	0.0 ± 0.0	1.0 ± 0.3	2.0 ± 1.0	3.0 ± 1.0*	2.6 ± 1.0*


VAS Hamstrings	Control	0.0 ± 0.0	1.1 ± 0.3	2.1 ± 0.5	4.2 ± 0.6*	2.4 ± 0.5*

(cm)	Pre	0.0 ± 0.0	0.7 ± 0.7	2.3 ± 0.7	3.4 ± 0.8*	2.4 ± 0.7

	Post	0.0 ± 0.0	1.2 ± 0.5	2.2 ± 0.7	4.4 ± 0.9*	4.1 ± 0.8*

CK	Control	165 ± 36	398 ± 95	623 ± 140	1032 ± 141*	801 ± 204

(U/L)	Pre	201 ± 51	382 ± 135	551 ± 135	853 ± 396	1098 ± 289*

	Post	199 ± 51	430 ± 134	759 ± 199	1531 ± 937*	937 ± 289*

LDH	Control	113 ± 7	125 ± 10	131 ± 18	186 ± 29	372 ± 78*

(U/L)	Pre	144 ± 10	154 ± 23	134 ± 25	159 ± 41	224 ± 110

	Post	124 ± 10	129 ± 22	129 ± 26	219 ± 41	419 ± 111*

Values are means ± standard deviation.

* *p *< 0.05, significantly different from 0 time point

Pre, received supplement before exercise and placebo after; Post, received supplement after exercise and placebo before exercise; Control, received placebo before and after exercise; CK, creatine kinase; MVC, maximal voluntary contraction; N, newtons; VAS, visual analogue scale.

## Discussion

This study was designed to determine if HMB taken in close proximity to exercise (acute effects) could lower indirect markers of muscle damage, and whether these acute effects were greater when the supplement was administered before as compared to after exercise (timing effects). Muscle damage was chemically inferred through plasma CK and LDH levels, functionally through isometric MVC of the quadriceps and hamstrings, and muscle soreness through the use of a VAS. Our study found that there were no clear differences in indirect markers of muscle damage regardless of the time of ingestion; however, we did notice that consuming HMB prior to exercise was able to prevent LDH values from significantly increasing following muscle damaging exercise.

We also noted that consuming HMB prior to exercise visually attenuated the rise in CK (Figure 3), and soreness in both the quadriceps (*p *= 0.07), and hamstrings (Figure 2). Intriguingly, the placebo and HMB-Post conditions experienced virtually identical changes in each of these parameters. In a similar cross over design study, Van Someren et al. [4] found that 2 weeks of HMB supplementation prior to muscle damaging exercise was able to significantly lower both serum markers of muscle damage and muscle soreness. Serum markers of muscle damage are known to be notoriously variable [16,24,25] and perhaps in order to have enough power to see significant effects of consuming HMB prior to exercise relative to the placebo and HMB-Post groups it may need to be loaded (≥ 2 weeks) prior to the exercise bout such as was done in the study by Van Someren et al. [4].

All of the studies that we are aware of have administered HMB for at least 2 weeks prior to the muscle damaging bout [3,6,11-13]. Presently, we are unaware of what the length of time needed for loading HMB would be in order to see significant effects. Exposure of isolated non muscle tissues to acid has revealed HMB concentrations as high as 100 μM [26]. If HMB is covalently bound to muscle tissue, it is conceivable that consuming the supplement increases these concentrations over time. With other amino acid derivatives such as creatine monohydrate, loading when administering large bolus ingestions (20 grams per day) can saturate muscle tissue within 1 week's time, while supplementing with lower doses (3 grams) takes longer to saturate muscle (28 days) [27]. If a period of loading is necessary, then it may explain the dose response seen in past studies. For example, Nissen et al.[3] found that consuming HMB at 3 grams per day while resistance training 3 days per week over 32 weeks time resulted in greater changes in total body strength and lean body mass than 1.5 grams. While this is only speculative, it may be that 3 grams resulted in greater saturation of muscle tissue with HMB than the 1.5 gram condition.

One limitation in our study would be that we analyzed indirect indices of muscle damage. However, there are a number of direct measures including z-line streaming, zigzagging, and centralized nuclei which may give valuable information as to the effects of HMB on indices of muscle damage.

In conclusion, our findings suggest that there were no clear acute or timing effects of HMB supplementation on indirect markers of muscle damage. However, consuming HMB an hour before exercise appeared to prevent increases in select markers of damage. It appears that HMB may need a loading period prior to exercise in order to significantly attenuate the rise in indices of muscle damage relative to placebo and HMB-Post conditions. Similar to creatine based studies, we suggest that future researchers examine how loading effects the concentration of HMB into skeletal muscle tissue, and what variables including duration of the loading period, and dosage loaded play in saturating skeletal muscle.

## Competing interests

The authors declare that they have no competing interests.

## Authors' contributions

Design and development of this trial conducted at Florida State University's nutrition, food and exercise sciences research laboratory was performed by JMW and LBP. Data collection was carried out by JMW, BD, JDK, HK, RS and S-RL. Blood processing and analysis of serum indices of damage were carried out by JMW, J-SK, RS, and S-RL. Statistical analysis, data evaluation, manuscript preparation and manuscript revisions were carried out by JMW, AM, J-SK, LBP, JDK, and JR. All authors read and approved the final manuscript.
